# One day versus two days of hepatic arterial infusion with oxaliplatin and fluorouracil for patients with unresectable hepatocellular carcinoma

**DOI:** 10.1186/s12916-022-02608-6

**Published:** 2022-10-31

**Authors:** Zhicheng Lai, Yexing Huang, Dongsheng Wen, Xuanjia Lin, Anna Kan, Qijiong Li, Wei Wei, Minshan Chen, Li Xu, Minke He, Ming Shi

**Affiliations:** Department of Hepatobiliary Oncology, Sun Yat-sen University Cancer Center, State Key Laboratory of Oncology in South China, Collaborative Innovation Center for Cancer Medicine, Guangzhou, 510060 China

**Keywords:** HAIC, Unresectable HCC, Treatment regimens, Thymidylate synthase, Biomarkers

## Abstract

**Background:**

Hepatic arterial infusion chemotherapy (HAIC) with oxaliplatin and 5-fluorouracil was effective in unresectable hepatocellular carcinoma (HCC). The program of FOLFOX-HAIC in HCC was performed for 1 day (HAIC 1d) or 2 days (HAIC 2d). We hereby retrospectively compared the efficacy and safety between these two treatment regimens and explored the predictive power of thymidylate synthase (TYMS), an enzyme involved in the DNA synthesis process and metabolism of fluorouracil.

**Methods:**

This study included patients with a primary diagnosis of unresectable HCC. These patients received HAIC for 1 day or 2 days. The overall survival (OS), progression-free survival (PFS), tumor response, and adverse events were compared. The propensity score matching (PSM) was used to reduce bias. Peripheral blood samples before the treatments were collected and used to measure the concentration of TYMS through enzyme-linked immunosorbent assay (ELISA). ELISA was performed according to the manufacturers’ guidelines.

**Results:**

We included 368 patients for this study: 248 in the HAIC 1d group and 120 in the HAIC 2d group. There was no significant difference of OS between the two groups (14.5 for HAIC 1d vs 15.3 months for HAIC 2d, *p*=0.46). Compared with the HAIC 1d group, the HAIC 2d group did not prolong the PFS (7.3 vs 7.5 months, *p*=0.91) or elevate the tumor response (42.5% vs 39.1%, *p*=0.53) per RECIST 1.1. In the PSM cohort, the efficacy between the two groups was similar. The total frequencies of grade 3–4 events were higher with the HAIC 2d group than with the HAIC 1d group, especially in the PSM cohort (*p*=0.043). Additionally, patients with TYMS low level might benefit longer OS from the HAIC 2d group (18.7 vs 13.6 months, *p*=0.014).

**Conclusions:**

There was not much of a difference in efficacy between the two groups, but the HAIC for 1 day might be safer, which needed further research. The level of TYMS might be the predictive biomarkers.

**Supplementary Information:**

The online version contains supplementary material available at 10.1186/s12916-022-02608-6.

## Background

Approximately half of hepatocellular carcinoma (HCC) patients are first diagnosed with unresectable disease, and the prognosis is poor [1–3]. Transcatheter arterial chemoembolization (TACE) or systemic therapies are the recommended first-line therapies for HCC with BCLC stage B or C, respectively [1, 4–6]. However, the efficacy of these therapies is still unsatisfactory for HCC with high-risk disease or disease beyond up-to-seven criteria [7, 8].

Recently, hepatic arterial infusion chemotherapy (HAIC) with oxaliplatin, fluorouracil, and leucovorin has shown promising anti-tumor activity for HCC with a high intrahepatic burden. The results from one phase 3 trial showed that compared with TACE, HAIC significantly improved the overall survival (OS) with a significantly lower incidence of grade 3–4 adverse events for large and unresectable HCC [9]. In 2019, another phase 3 trial showed that HAIC plus sorafenib was associated with a significant benefit in overall survival in advanced HCC with portal vein invasion (PVTT), in which more than 80% of enrollments had advanced PVTT (Vp3 or Vp4) [10]. More recently, the combination of HAIC with systemic therapy, such as lenvatinib and programmed cell death protein-1 (PD-1), has been suggested to significantly improve the prognosis of advanced HCC [11–13]. HAIC is now accepted as a treatment option for unresectable HCC and is promoted in the clinic [14–16].

Until now, the regimens of HAIC have not been unified. The HAIC program in HCC is usually performed with oxaliplatin, leucovorin, fluorouracil bolus on day 1, and fluorouracil infusion for 2 days [14, 17–20]. Although the HAIC program for 2 days is effective, the activity restriction for patients was up to 2 days, which increased the cost of hospitalization, reduced patients’ health care compliance, and potentially caused new health problems such as lower extremity deep venous thrombosis. Therefore, some investigators have shortened the fluorouracil infusion time to 1 day, which might help maintain higher blood concentrations in the liver to improve the efficacy [9, 11, 15, 21]. However, no published studies have directly compared these two dosing regimens. Additionally, thymidylate synthase (TYMS) is an enzyme involved in the DNA synthesis process and metabolism of fluorouracil [22]. Previous studies demonstrated that the mRNA levels of TYMS are related to the response to fluorouracil [23]. Nevertheless, it is not clear whether the peripheral serum level of TYMS interacts with the anti-tumor activity of different fluorouracil infusion times.

Therefore, we retrospectively compared the efficacy and safety of HAIC for 1 day to HAIC for 2 days and explored the role of the peripheral serum level of TYMS between the two treatment groups.

## Methods

### Patients

This retrospective study was conducted following the International Conference on Harmonisation guidelines for Good Clinical Practice and the principles of the Declaration of Helsinki at Sun Yet-sen University Cancer Center in China. The study was approved by the institutional review board and the ethics committee (B2022-114-01). All patients gave written informed consent. Resectability was assessed by the same 2 experienced liver surgeons in our hospitals. Resectable disease was defined as the complete removal of all macroscopic tumor tissue, portal vein tumor thrombus, and hepatic vein tumor thrombus with an expected remnant liver volume no less than 250 ml/m^2^. Once a diagnosis of unresectable HCC was confirmed, the patients were informed that HAIC was recommended based on previous studies [9, 10, 24, 25]. Patients with intermediate HCC were recommended HAIC monotherapy, while patients with advanced HCC were recommended HAIC plus sorafenib or lenvatinib.

Eligible patients were 18 years of age or older and had unresectable HCC, with the diagnosis confirmed by histologic or cytologic analysis or clinical features [1]. Eligible patients had not previously received treatment and had at least one measurable disease, as defined by Response Evaluation Criteria In Solid Tumours 1.1 (RECIST 1.1) criteria [26], no cirrhosis or cirrhotic status of Child-Pugh class A only, and adequate hematologic and organ function (absolute neutrophil count ≥1.2×10^9^/l, platelet count ≥60×10^9^/l, total bilirubin <30μmol/l, albumin ≥30g/l, aspartate transaminase and alanine transaminase ≤5×upper limit of the normal, creatinine clearance rate of ≤1.5×upper limit of the normal, and left ventricular ejection ≥45%). Among the key exclusion criteria were history of HIV, organ allograft, combined with other malignant tumors, evidence of hepatic decompensation, bleeding diathesis or event, and allergy to the investigational agents or any agent given in association with this trial and incomplete medical information.

### Treatments

HAIC treatment was divided into 3-week cycles. The microcatheter was advanced into the hepatic artery according to our previous studies [9, 10]. And patients were transferred to the inpatient ward for drug infusion via the hepatic artery. Oxaliplatin, leucovorin, and bolus fluorouracil were conducted equally in both groups, while infusional fluorouracil 2400 mg/m^2^ was given over 46 h in the HAIC for 2 days group (HAIC 2d) and over 23 h in the HAIC for 1 day group (HAIC 1d), respectively. After HAIC was completed, the catheter and sheath were removed. Repetitive femoral artery puncture and catheterization were performed in the next HAIC cycle.

### Outcomes

The OS, progression-free survival (PFS), objective response rate (ORR), disease control rate (DCR), and adverse events were compared between the two groups. OS was defined as the time from the commencement of treatment to death from any cause or the date of the last follow-up if the patient was alive. PFS was the interval from the commencement of treatment to disease progression according to RECIST 1.1 or death from any cause, whichever occurred first. ORR was the proportion of patients with complete response or partial response that was maintained for at least 4 weeks from the first radiological confirmation, and DCR was the proportion of patients with ORR plus stable disease [26, 27]. Adverse events were evaluated by vital signs and clinical laboratory test results and assessment of the incidence and severity of adverse events according to the National Cancer Institute [NCI] Common Terminology Criteria for Adverse Events, version 4.0.

### ELISA

Peripheral serum before the treatment was used for quantitative detection of TYMS using a commercial ELISA kit. The details are provided in Additional file 1: Methods.

### Statistical analysis

Propensity score matching (PSM) analysis was conducted to reduce the influence of selection bias. The following parameters were included in the PSM: absence or presence of PVTT, absence or presence of HVTT, absence or presence of metastasis, tumor size, tumor number, and AFP. Matched pairs were then formed using a 1-to-1 nearest-neighbor caliper width of 0.1.

We used SPSS (version 25.0) for all analyses. The results are reported as the mean (standard deviation [SD]), number (%), or median (95% confidence interval [CI]) and were compared by Student’s *t*-tests or chi-square tests. We set the difference at the upper limit of the CI to 3 months for OS and PFS to determine the much of difference. Hospitalization time was calculated from the beginning of HAIC treatment to discharge. The OS and PFS with associated 95% CIs were analyzed by the Kaplan–Meier method and were compared between treatment groups with the use of a log-rank test, and hazard ratios for disease progression or death were estimated with a Cox proportional hazards model. All *p* values were two-sided, with *p* values less than 0.05 considered significant.

## Results

**Fig. 1:**
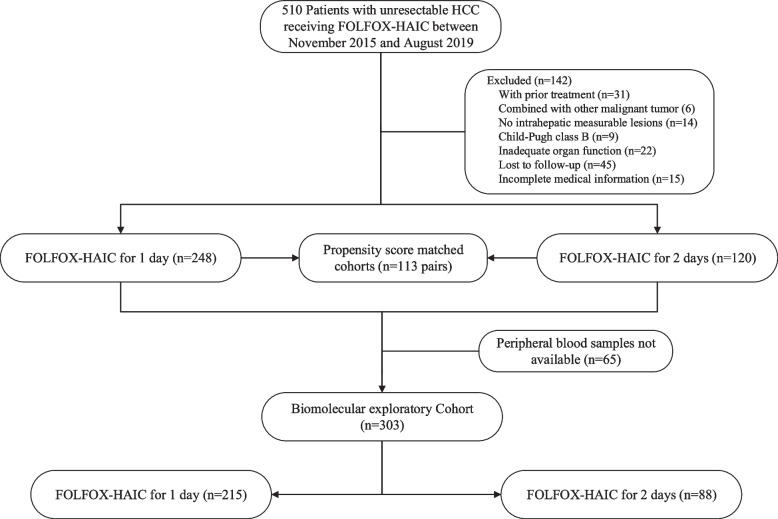
Flow diagram. FOLFOX, oxaliplatin, 5-fluorouracil, and leucovorin; HAIC, hepatic arterial infusion chemotherapy; HCC, hepatocellular carcinoma

Between November 27, 2015, and August 28, 2019, a total of 368 patients met the criteria for inclusion in this study: 120 patients received HAIC 2d, and 248 patients received HAIC 1d (Fig. 1). The follow-up went to December 19, 2021. The median tumor diameter was 10cm and the main etiology of HCC was hepatitis B virus (HBV) infection (88.9%). The patients with HBV infection all received antiviral therapy during the treatment. There were 245 of 368 patients (66.6%) with BCLC stage C in our study, and only 155 of 245 patients (63.3%) received tyrosine kinase inhibitors (TKIs) or immune checkpoint inhibitors (ICIs). There was no significant difference in systemic treatments between the two groups. After PSM, we obtained one-to-one paired cohorts (113 patients in each group). The median hospitalization time was 3.5 days for HAIC 1d and 5.3 days for HAIC 2d (*p*<0.0001). The baseline characteristics are shown in Table 1, and there was no significant difference between the two groups.

**Table 1:** Baseline characteristics

	HAIC 2d (*n*=120)	HAIC 1d(*n*=248)	*p*	PSM cohort		*p*
				HAIC 2d (*n*=113)	HAIC 1d(*n*=113)	
Age, year, mean (SD)	50.2 (11.8)	50.4 (11.9)	0.92	50.4 (11.7)	51.0 (11.6)	0.70
≤50	60	123	0.94	57	55	0.79
>50	60	125		56	58	
Sex			0.55			0.65
Male	107	226		101	103	
Female	13	22		12	10	
HbsAg			0.40			0.47
Positive	109	218		102	105	
Negative	11	30		11	8	
ALB, median (IQR), g/dL	41.3 (37.8–44.2)	40.6 (37.8–43.5)	0.25	41.2 (37.6–44.1)	40.9 (37.4–43.6)	0.34
ALT, median (IQR), U/L	42.2 (29.3–68.9)	44.9 (31.7–68.9)	0.27	44.3 (29.7–71.4)	45.9 (31.7–68.4)	0.31
AST, median (IQR), U/L	66.2 (46.6–103.5)	59.5 (41.6–101.7)	0.47	67.6 (48.9–111.25)	60.7 (41.9–106.2)	0.71
TBIL, median (IQR), μmol/L	15.7 (11.5–21)	15.6 (12–21.8)	0.83	15.9 (11.6–21)	16 (12.1–21.8)	0.61
PT, median (IQR), s	12.5 (11.8–13.4)	12.4 (11.7–13)	0.78	12.5 (11.8–13.4)	12.3 (11.7–13)	0.33
Tumor size, median (IQR), cm	9.6 (7.3–12.4)	10.2 (8.2–13.0)	0.09	9.7 (7.5–12.8)	9.8 (7.4–12.9)	0.67
≤10	68	118	0.10	61	58	0.69
>10	52	130		52	55	
Tumor number			0.32			0.34
≤3	50	90		43	50	
>3	70	158		70	63	
PVTT (Japan)			0.87			1
Vp1-2&No	72	151		65	65	
Vp3-4	48	97		48	48	
HVTT			0.78			0.79
No	100	199		94	90	
Hepatic vein	14	34		13	16	
Inferior vena cava	6	15		6	7	
Extrahepatic metastasis			0.74			0.45
No	92	180		85	74	
Organ only	10	24		10	15	
Lymph node only	14	30		14	18	
Both	4	14		4	6	
PIVKA-II, median (IQR), mAU/mL	7798 (569.1–38031.8)	8464.5 (1153.8–54135.3)	0.34	8233 (548–37792.50	6053 (826–53782.5)	0.57
CA199, median (IQR), U/ml	31.2 (18.0–53.2)	30.2 (14.5–55.1)	0.22	31.1 (18.4–52.6)	31.8 (12.8–56.2)	0.33
AFP, ng/mL			0.92			0.69
≤400	50	102		49	46	
>400	70	146		64	67	
BCLC stage			0.83			0.67
A or B	41	82		37	34	
C	79	166		76	79	
Combination therapy			0.69			0.90
No	69	139		73	73	
TKIs	47	104		37	38	
TKIs+ICIs	4	5		3	2	

*Abbreviations*: *AFP*, alpha-fetoprotein; *ALT*, alanine aminotransferase; *AST*, aspartate aminotransferase; *ALB*, albumin; *BCLC*, Barcelona Clinic Liver Cancer; *HbsAg*, hepatitis B surface antigen; *HVTT*, hepatic vein tumor thrombus; *ICIs*, immune checkpoint inhibitors; *PSM*, propensity score matching; *PVTT*, portal vein invasion; *TBIL*, total bilirubin; *TKIs*, tyrosine kinase inhibitors

Treatment administration is listed in Additional file 2: Table S1. After the administration of HAIC, the patients received second-line therapy, such as ablation (*p*=0.34), TACE (*p*=0.83), radiotherapy (*p*=0.26), sorafenib (*p*=0.74), lenvatinib (*p*=0.43), and PD-1 antibody (*p*=0.30). Additionally, subsequent surgical resection was conducted for 16 patients in the HAIC 2d group and 32 patients in the HAIC 1d group (*p*=0.91).

### Efficacy

**Fig. 2:**
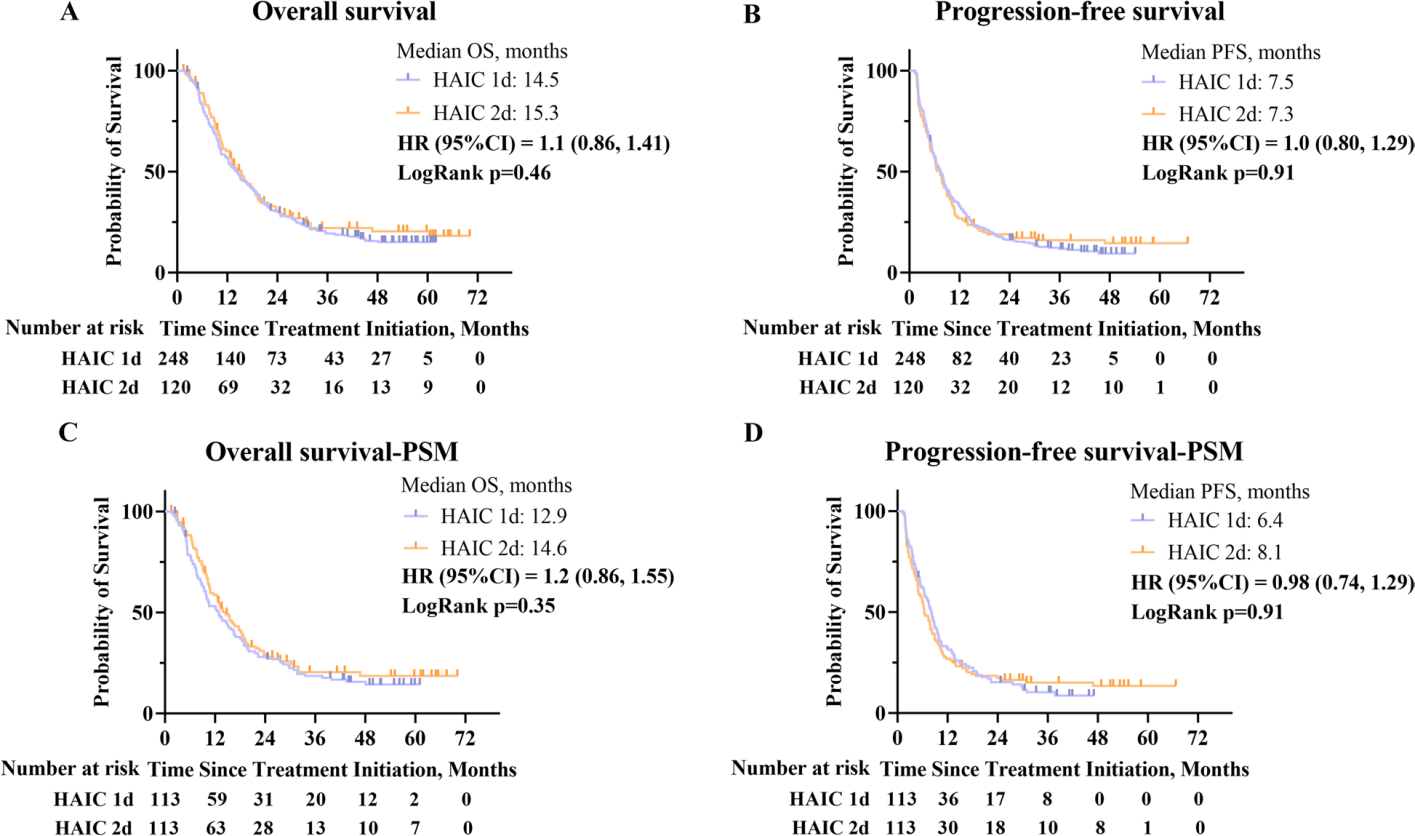
Kaplan–Meier curves of overall survival (**A**), progression-free survival (**B**), overall survival for the PSM cohort (**C**), and progression-free survival for the PSM cohort (**D**). CI, confidence interval; HR. hazard ratio; PSM, propensity score matching

The median OS of the HAIC 1d group (14.5 months, 95% CI, 11.9–17.0) suggested no significant difference compared with that of the HAIC 2d group (15.3 months, 95% CI, 12.4–18.1) (*p*=0.46) (Fig. 2A). Similarly, there was no significant difference between the PFS of the HAIC 1d group (7.5 months, 95% CI, 6.4–8.6) and that of the HAIC 2d group (7.3 months, 95% CI, 5.9–8.7) (*p*=0.91) (Fig. 2B). The difference of the upper limit of the CI between the two groups was 1.1 months for OS and 0.1 months for PFS. Furthermore, the OS and PFS also had no significant difference between the HAIC 1d group and the HAIC 2d group in BCLC stage C patients across different combination treatment subgroup (Additional file 3: Fig. S1).

The results of univariate and multivariate analyses of OS and PFS are listed in Additional file 2: Table S2. The treatment group was not an independent risk factor for OS (HR=1.1, 95% CI, 0.86–1.41, *p*=0.46) or PFS (HR=1.0, 95% CI, 0.80–1.28, *p*=0.91). Multivariate analysis showed that the independent risk factors for OS were tumor size (>10 cm vs. ≤10 cm, HR=1.3; 95% CI, 1.1–1.7; *p*=0.019), tumor number (>3 vs. ≤3, HR=1.5; 95% CI, 1.1–1.9; *p*<0.001), PVTT (Vp3-4 vs. Vp1-2 and none, HR=1.6; 95% CI, 1.2–2.0; *p*<0.001), metastasis (presence vs. absence, HR=1.9; 95% CI, 1.5–2.5; *p*<0.001), AFP (>400 vs. ≤400, HR=1.4; 95% CI, 1.1–1.8; *p*=0.01), and ALBI (grade 2 vs. grade 1, HR=1.5; 95% CI, 1.2–1.9; *p*<0.001). The independent risk factors for PFS were tumor number (>3 vs. ≤3, HR=1.4; 95% CI, 1.1–1.8; *p*=0.008) and metastasis (presence vs. absence, HR=1.9; 95% CI, 1.4–2.4; *p*<0.001).

**Fig. 3:**
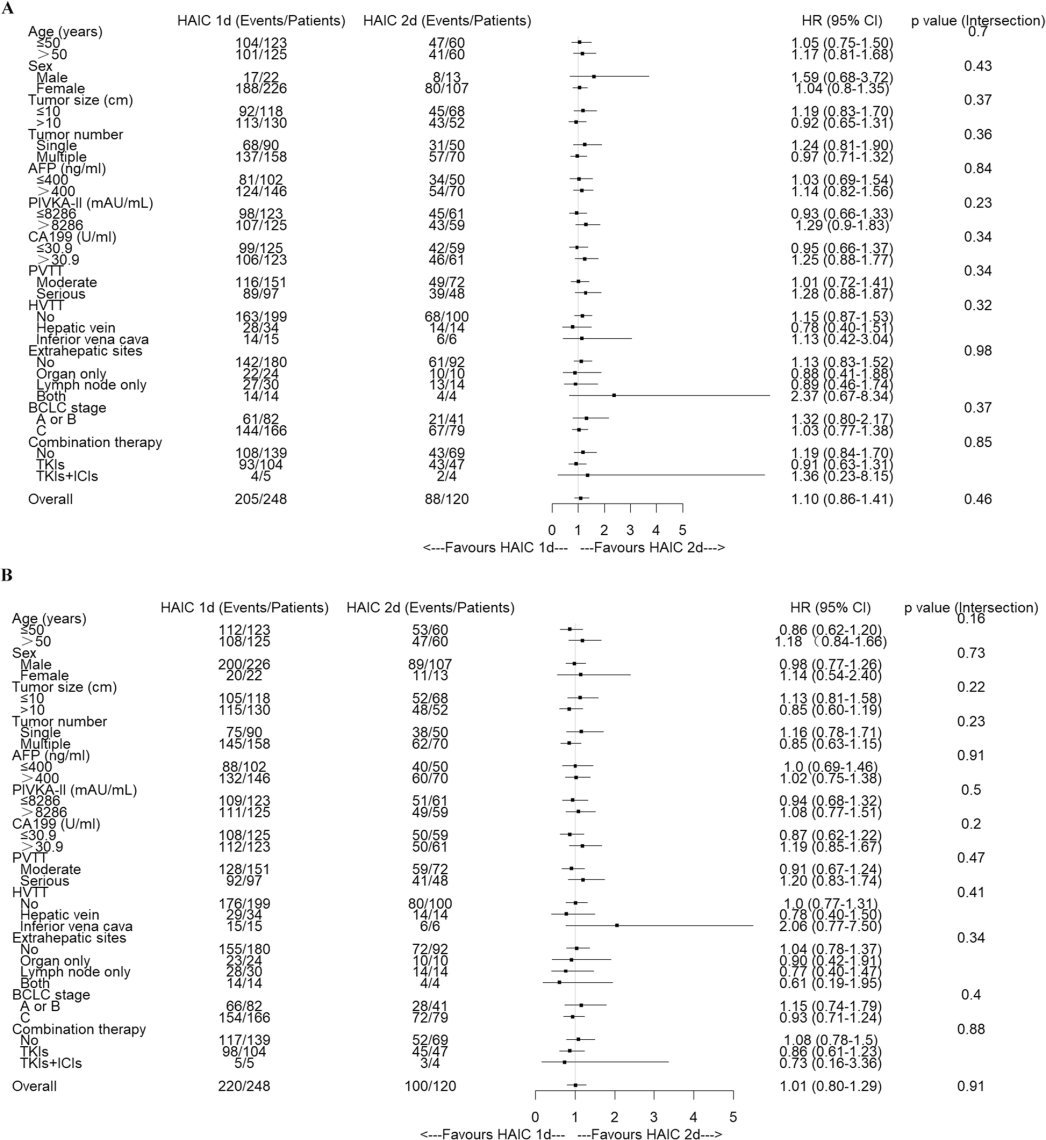
Forest plot of factors associated with overall survival (**A**) and progression-free survival (**B**) in patients treated with HAIC 1d versus HAIC 2d

The OS and PFS benefits with HAIC 1d compared with HAIC 2d across the clinically relevant subgroups are shown in Fig. 3A, B. Prolonging the fluorouracil infusion time did not provide any clinical benefit for OS and PFS in all subgroups.

The tumor response rate of the patients is shown in Table 2. The confirmed objective response rate (ORR) was 39.1% in the HAIC 1d group and 42.5% in the HAIC 2d group per RECIST1.1 (*p*=0.53) and 41.5% and 50% per mRECIST (*p*=0.13). Similarly, the DCR based on RECIST1.1 or mRECIST criteria was not significantly different between the HAIC 1d group and the HAIC 2d group (*p*=0.75).

**Table 2:** Tumor response

	RECIST 1.1			mRECIST		
	HAIC 2d	HAIC 1d	*p*^a^	HAIC 2d	HAIC 1d	*p*^a^
CR	0	0		7 (5.8%)	13 (5.2%)	0.82
PR	51 (42.5%)	97 (39.1%)	0.53	53 (44.2%)	90 (36.3%)	0.15
SD	37 (30.8%)	81 (32.7%)	0.73	28 (23.3%)	74 (29.8%)	0.19
PD	22 (18.3%)	48 (19.4%)	0.82	22 (18.3%)	49 (19.8%)	0.75
NA	10 (8.3%)	22 (8.9%)	0.86	10 (8.3%)	22 (8.9%)	0.86
ORR	51 (42.5%)	97 (39.1%)	0.53	60 (50.0%)	103 (41.5%)	0.13
DCR	88 (73.3%)	178 (71.8%)	0.75	88 (73.3%)	178 (71.8%)	0.75
	RECIST 1.1			mRECIST		
PSM cohort	HAIC 2d	HAIC 1d	*p*^a^	HAIC 2d	HAIC 1d	*p*^a^
CR	0	0		7 (6.2%)	4 (3.5%)	0.35
PR	45 (39.8%)	41 (36.3%)	0.58	47 (41.6%)	42 (37.2%)	0.50
SD	37 (32.7%)	39 (34.5%)	0.78	28 (27.2%)	34 (30.1%)	0.64
PD	21 (18.6%)	21 (18.6%)	1	21 (18.6%)	21 (18.6%)	1
NA	10 (8.8%)	12 (10.6%)	0.65	10 (8.8%)	12 (10.6%)	0.65
ORR	45 (39.8%)	41 (36.3%)	0.58	54 (47.8%)	46 (40.7%)	0.28
DCR	82 (72.6%)	80 (70.8%)	0.77	82 (72.6%)	80 (70.8%)	0.77

*Abbreviations*: *CR*, complete response; *DCR*, disease control rate; *mRECIST*, modified Response Evaluation Criteria in Solid Tumors; *NA*, not assessable; *ORR*, objective response rate; *PD*, progressive disease; *PR*, partial response; *RECIST*, Response Criteria in Solid Tumors; *SD*, stable disease

^a^Statistical significance was assessed with the chi-square test

In the PSM cohort, the median OS of the HAIC 1d group (12.9 months, 95% CI, 9.1–16.8) was also not inferior to that of the HAIC 2d group (14.6 months, 95% CI, 11.6–17.5) (*p*=0.35) (Fig. 2C). The median PFS in the HAIC 1d group was 8.1 months (95% CI, 6.8–9.3) compared with 6.4 months (95% CI, 4.9–8.0) in the HAIC 2d group (*p*=0.87) (Fig. 2D). For BCLC stage C patients, the OS and PFS had no significant difference between the two groups across the different combination treatment subgroups (Additional file 4: Fig. S2). Similarly, the treatment group was not an independent risk factor for OS or PFS. HbsAg, tumor number, HVTT, metastasis, AFP, and ALBI were independent risk factors for OS, and tumor number, PVTT, HVTT, and metastasis were independent risk factors for PFS (Additional file 2: Table S3). Except for patients with an involved inferior vena cava, prolonging the infusion time of fluorouracil did not provide clinical benefits for OS and PFS in all subgroups (Additional file 5: Fig. S3A&B). Additionally, the ORR (36.3% vs. 39.8%, *p*=0.58) and DCR (70.8% vs. 72.6%, *p*=0.77) according to RECIST 1.1 criteria were not significantly different between the two groups (Table 2).

### Safety

There were no treatment-related deaths in this study, and the treatment-related AEs with high incidence rates (≥10%) are shown in Table 3. The frequencies of grade 3–4 diarrhea (5 [3.8%] vs. 0 [0%]; *p*=0.003), elevated alanine aminotransferase (13 [10.5%] vs. 11 [4.4%]; *p*=0.02), and elevated aspartate aminotransferase (40 [33.3%] vs. 52 [21%]; *p*=0.01) were significantly higher in the HAIC 2d group than in the HAIC 1d group. However, the total frequencies of grade 3–4 events were not significantly different between the HAIC 2d group (56 patients [46.7%]) and the HAIC 1d group (90 patients [36.3%]) (*p*=0.056). In addition, the frequencies of all-grade fatigue (*p*<0.001), sensory neuropathy (*p*<0.001), alopecia (*p*<0.001), nausea (*p*=0.001), vomiting (*p*=0.044), diarrhea (*p*=0.015), neutropenia (*p*=0.001), anemia (*p*=0.048), thrombocytopenia (*p*=0.027), elevated aspartate aminotransferase (*p*=0.023), and prolonged PT (*p*<0.001) were significantly higher in the HAIC 2d group, while the frequency of all-grade elevated creatinine was significantly higher in the HAIC 1d group (*p*=0.002). In addition, upper gastrointestinal bleeding was observed in 2 patients in the HAIC 1d group, and the patients recovered under medical treatment.

**Table 3:** Treatment-related adverse events

	HAIC 2d (*n*=120)				HAIC 1d (*n*=248)				*p*	*p* for grade 3–4 AE
	Any grade	Grades 1–2	Grade 3	Grade 4	Any grade	Grades 1–2	Grade 3	Grade 4		
Hypertension	44 (36.7%)	43 (36.2%)	1 (0.95%)	0	83 (33.5%)	79 (31.9%)	4 (1.6%)	0	0.55	1
Fatigue	87 (72.4%)	87 (72.4%)	0	0	99 (39.9%)	98 (39.5%)	1 (0.4%)	0	<0.001	1
Fever	16 (13.3%)	16 (13.3%)	0	0	19 (7.7%)	19 (7.7%)	0	0	0.082	
Sensory neuropathy	46 (38.1%)	46 (38.1%)	0	0	28 (11.3%)	27 (10.9%)	1 (0.4%)	0	<0.001	1
Edema	15 (12.4%)	15 (12.4%)	0	0	21 (8.5%)	17 (6.9%)	4 (1.6%)	0	0.22	0.31
Alopecia	23 (19.1%)	23 (19.1%)	0	0	10 (4.0%)	10 (4.0%)	0	0	<0.001	
Abdominal pain	70 (58.1%)	69 (57.1%)	1 (0.95%)	0	128 (51.6%)	121 (48.8%)	5 (2.0%)	2 (0.8%)	0.23	0.4
Nausea	91 (76.2%)	89 (74.3%)	2 (1.9%)	0	140 (56.5%)	137 (55.2%)	3 (1.2%)	0	<0.001	0.66
Vomit	49 (41.0%)	41 (34.3%)	8 (6.7%)	0	75 (30.2%)	61 (24.6%)	14 (5.7%)	0	0.044	0.7
Diarrhea	31 (25.7%)	26 (21.9%)	5 (3.8%)	0	38 (15.3%)	38 (15.3%)	0	0	0.015	0.003
Neutropenia	21 (17.5%)	19 (16.2%)	1 (0.95%)	1 (0.95%)	16 (6.5%)	13 (5.2%)	2 (0.8%)	1 (0.4%)	0.001	0.66
Anemia	83 (69.5%)	82 (68.6%)	1 (0.95%)	0	145 (58.5%)	145 (58.5%)	0	0	0.048	0.33
Thrombocytopenia	58 (48.6%)	56 (46.7%)	2 (1.9%)	0	90 (36.3%)	75 (30.2%)	10 (4.0%)	5 (2.0%)	0.027	0.06
Elevated ALT	84 (70.0%)	71 (59.1%)	13 (10.5%)	0	160 (64.5%)	149 (60.1%)	11 (4.4%)	0	0.30	0.02
Elevated AST	119 (99.1%)	79 (65.7%)	37 (30.5%)	3 (2.9%)	237 (95.6%)	185 (74.6%)	48 (19.4%)	4 (1.6%)	0.13	0.01
Hyperbilirubinemia	57 (47.6%)	56 (46.7%)	1 (0.95%)	0	98 (39.5%)	96 (38.7%)	2 (0.8%)	0	0.15	1
Elevated CRE	11 (9.5%)	11 (9.5%)	0	0	56 (22.6%)	56 (22.6%)	0	0	0.002	
Hypoalbuminemia	109 (90.5%)	109 (90.5%)	0	0	233 (94.0%)	233 (94.0%)	0	0	0.27	
Prolonged PT	57 (47.6%)	57 (47.6%)	0	0	63 (25.4%)	63 (25.4%)	0	0	<0.001	
Grades 3–4			56 (46.7%)				90 (36.3%)			0.056

In the PSM cohort, the total frequencies of grade 3–4 events were significantly higher in the HAIC 2d group than in the HAIC 1d group (*p*=0.043) (Table 4). The frequencies of grade 3–4 elevated alanine aminotransferase and elevated aspartate aminotransferase and the frequencies of any grade fatigue, fever, sensory neuropathy, alopecia, nausea, elevated aspartate aminotransferase, and prolonged PT were also significantly higher in the HAIC 2d group, while the frequency of any grade elevated creatine was also significantly higher in the HAIC 1d group.

**Table 4:** Treatment-related adverse events in the propensity score-matched cohort

	HAIC 2d (*n*=113)				HAIC 1d (*n*=113)				*p*	*p* for grade 3–4 AE
	Any grade	Grades 1–2	Grade 3	Grade 4	Any grade	Grades 1–2	Grade 3	Grade 4		
Hypertension	40 (35.4%)	39 (34.5%)	1 (0.9%)	0	46 (40.7%)	44 (38.9%)	2 (1.8%)	0	0.41	1.0
Fatigue	80 (70.8%)	80 (70.8%)	0	0	45 (39.8%)	44 (38.9%)	1 (0.9%)	0	<0.001	1.0
Fever	16 (14.2%)	16 (14.2%)	0	0	4 (3.5%)	4 (3.5%)	0	0	0.005	
Sensory neuropathy	42 (37.2%)	42 (37.2%)	0	0	12 (10.6%)	11 (9.7%)	1 (0.9%)	0	<0.001	1.0
Edema	15 (13.3%)	15 (13.3%)	0	0	8 (7.1%)	7 (6.2%)	1 (0.9%)	0	0.12	1.0
Alopecia	23 (20.4%)	23 (20.4%)	0	0	4 (3.5%)	4 (3.5%)	0	0	<0.001	
Abdominal pain	64 (56.6%)	63 (55.8%)	1 (0.9%)	0	60 (53.1%)	57 (50.4%)	2 (1.8%)	1 (0.9%)	0.59	0.62
Nausea	84 (74.3%)	82 (72.6%)	2 (1.8%)	0	62 (54.9%)	59 (52.2%)	3 (2.7%)	0	0.002	1.0
Vomit	46 (40.7%)	39 (34.5%)	7 (6.2%)	0	33 (29.2%)	29 (25.7%)	4 (3.5%)	0	0.07	0.35
diarrhea	29 (25.7%)	24 (21.2%)	5 (4.4%)	0	19 (16.8%)	19 (16.8%)	0	0	0.10	0.06
Neutropenia	21 (18.6%)	19 (16.8%)	1 (0.9%)	1 (0.9%)	12 (10.6%)	10 (8.9%)	2 (1.8%)	0	0.09	1.0
Anemia	79 (69.9%)	78 (69.0%)	1 (0.9%)	0	66 (58.4%)	66 (58.4%)	0	0	0.071	1.0
Thrombocytopenia	56 (49.6%)	54 (47.8%)	2 (1.8%)	0	45 (39.8%)	38 (33.6%)	4 (3.5%)	3 (2.7%)	0.14	0.17
Elevated ALT	81 (71.7%)	68 (60.2%)	13 (11.5%)	0	73 (64.6%)	68 (60.2%)	5 (4.4%)	0	0.25	0.049
Elevated AST	112 (99.2%)	73 (64.6%)	37 (32.7%)	2 (1.8%)	105 (92.9%)	82 (72.6%)	20 (17.7%)	3 (2.7%)	0.041	0.017
Hyperbilirubinemia	55 (48.7%)	54 (47.8%)	1 (0.9%)	0	50 (44.3%)	48 (42.5%)	2 (1.8%)	0	0.51	1.0
Elevated CRE	11 (9.7%)	11 (9.7%)	0	0	27 (23.9%)	27 (23.9%)	0	0	0.004	
Hypoalbuminemia	104 (92.0%)	104 (92.0%)	0	0	104 (92.0%)	104 (92.0%)	0	0	1.000	
Prolonged PT	55 (48.7%)	55 (48.7%)	0	0	29 (25.7%)	29 (25.7%)	0	0	<0.001	
Grade 3–4 AE			55 (48.7%)				40 (35.4%)			0.043

### Patients with peripheral serum low levels of TYMS benefited from HAIC 2d

We performed ELISA to quantify the concentration of TYMS in peripheral serum from a total of 303 patients. The baseline characteristics and tumor response of the 303 patients are shown in Additional file 2: Table S5, and there was no significant difference between the two treatment groups.

**Fig. 4:**
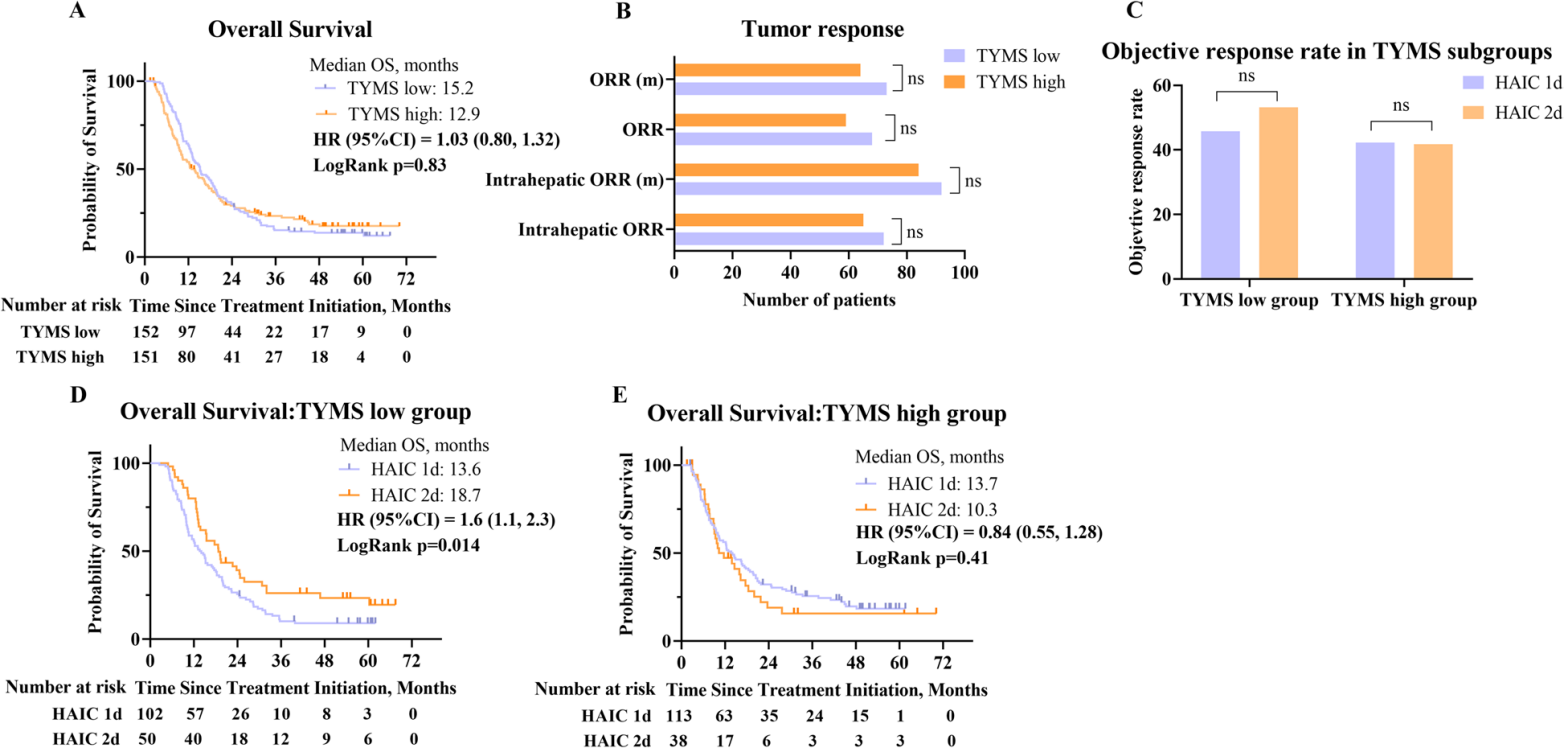
Correlation between TYMS level and efficacy. **A** Kaplan–Meier curve of overall survival between the TYMS low group and the TYMS high group. **B** The tumor response between the TYMS low group and the TYMS high group. m, mRECIST. **C** The tumor response between the HAIC 1d group and the HAIC 2d group within the TYMS low group and the TYMS high group. **D** Kaplan–Meier curve of overall survival between HAIC 1d and HAIC 2d within the TYMS low group. **E** Kaplan–Meier curve of overall survival between HAIC 1d and HAIC 2d within the TYMS high group. Patients were divided into the TYMS high group and the TYMS low group according to the median level of TYMS from 303 patients. mRECIST, modified Response Evaluation Criteria in Solid Tumors; ORR, objective response rate; RECIST, Response Criteria in Solid Tumors; TYMS, thymidylate synthase

The median concentration of TYMS in the HAIC 2d group was 3.6 ng/μl, compared with 4.1 ng/μl in the HAIC 1d group (*p*=0.16). We divided the patients into the TYMS high group and the TYMS low group according to the median concentration of TYMS in 303 patients. The OS was not significantly different between the TYMS high group and the TYMS low group (12.9 vs. 15.2 months, *p*=0.83) (Fig. 4A). The ORR per RECIST 1.1 or mRECIST was not significantly different between the two groups (Fig. 4B). Furthermore, neither in the TYMS low group nor in the TYMS high group, the ORR had no significant difference between HAIC 1d group and HAIC 2d group (Fig. 4C). Interestingly, within the TYMS low group, patients in the HAIC 2d group had significantly longer OS than those in the HAIC 1d group (18.7 vs. 13.6 months, *p*=0.014) (Fig. 4D). Similarly, patients in the HAIC 1d group had longer OS without a significant difference within the TYMS high group (13.7 vs. 10.3 months, *p*=0.41) (Fig. 4E).

## Discussion

This is the first study to compare HAIC 1d with HAIC 2d for intermediate or advanced HCC. The results from this study suggested that the OS, PFS, and tumor response rates of patients with unresectable HCC did not differ significantly between HAIC 1d and HAIC 2d. And it also suggested no evidence of difference for OS or PFS could be as much as 3 months or 1 month between the two groups. Patients with low TYMS levels might benefit, with a longer OS, from the HAIC 2d regimen. In addition, the treatment groups were not independent risk factors for OS or PFS. However, the total frequencies of grade 3–4 events were higher but not significantly different in the HAIC 2d group than in the HAIC 1d group (*p*=0.056). In the PSM cohort, the efficacy between the two groups was also not significantly different, and the total frequencies of grade 3–4 events were significantly higher with the HAIC 2d group than with the HAIC 1d group (*p*=0.043).

The anti-tumor activity of HAIC majorly depends on the tumor local concentration and infusion time. The results from the pharmacokinetics of fluorouracil following HAIC in a VX2 hepatic metastasis model showed that shortening the fluorouracil infusion time significantly increased the tumor local concentration at the same dose [28]. Our results suggested that the prognosis of HAIC 2d was significantly better than that of HAIC 1d within patients with low TYMS levels. The possible reason was that low-flow infusion might still achieve effective anti-tumor concentrations in these patients, while prolonged infusion time further enhanced the anti-tumor activity. Therefore, patients with TYMS low level on HAIC 2d regimen showed the best ORR and OS. On the other hand, the prognosis had no significant difference between HAIC 2d and HAIC 1d within patients with TYMS high level. We thought that it might be due to the accelerated metabolism of fluorouracil, which maintained a lower local concentration in low-flow infusion, thereby attenuating the anti-tumor activity.

A subanalysis of OS and PFS was performed based on various factors. Our results suggested that prolonging the fluorouracil infusion time did not provide clinical benefits for OS and PFS in most subgroups. Although patients with an involved inferior vena cava benefited in terms of PFS from the HAIC 2d group in the PSM cohort (HR, 8.7, 95% CI, 1.7–44.3), we thought that this was due to bias caused by the small sample size.

Although the anti-tumor activity was similar between the two groups, the total frequencies of grade 3–4 events in the HAIC 2d group were higher than those in the HAIC 1d group. Fluorouracil could impair liver function through cholestasis, and our results suggested that prolonging the fluorouracil infusion time further impaired liver function. Additionally, our results also suggested that prolonging the fluorouracil infusion time increased the incidence of gastrointestinal side effects and bone marrow suppression. However, shortening the fluorouracil infusion time to 1 day increased the incidence of renal impairment, which might be due to kidney filtration of a high concentration of fluorouracil in a short time. In general, these adverse events were expected and manageable by treatment interruption or dose modification.

This study had several limitations. First, this was a retrospective study performed at a single medical site, which might limit the interpretation of the results. However, the baseline characteristics were well balanced between the two groups, and PSM analysis was used to further improve the comparability. Second, the expression and polymorphisms of several genes, such as TYMS, DPYD, and MTHFR, are involved together in the metabolism of fluorouracil [29–31]. .However, it was unclear whether the mutation profile of the above genes was significantly different between the two treatment groups. Third, there was a lack of solid pharmacokinetic evidence for the local concentration in HAIC and a clinically applicable novel technique for measuring the drug concentration in the liver is needed.

## Conclusions

There was not much of a difference in efficacy between the HAIC 1d group and the HAIC 2d group, but the HAIC for 1 day might be safer, which needed further research. The level of TYMS might be the predictive biomarkers for patients who underwent HAIC.

## Supplementary Information

**Additional file 1.** Methods. The details of ELISA.

**Additional file 2: Table S1.** Treatment administration. **Table S2.** Univariate and multivariate analysis of overall survival and progression-free survival. **Table S3.** Univariate and multivariate analysis of overall survival and progression-free survival in the PSM cohort. **Table S4.** Intrahepatic tumor response. **Table S5.** Baseline characteristics and tumor response within patients performing ELISA detection.

**Additional file 3: Figure S1.** Kaplan-Meier curves of overall survival in BCLC stage C patients receiving HAIC alone (A), HAIC + TKIs (B) and HAIC + TKIs + ICIs (C). Kaplan-Meier curves of progression-free survival in BCLC stage C patients receiving HAIC alone (D), HAIC + TKIs (E) and HAIC + TKIs + ICIs (F). CI, confidence interval; HR. hazard ratio.

**Additional file 4: Figure S2.** Kaplan-Meier curves of overall survival in BCLC stage C patients receiving HAIC alone (A), HAIC + TKIs (B) and HAIC + TKIs + ICIs (C) in PSM cohort. Kaplan-Meier curves of progression-free survival in BCLC stage C patients receiving HAIC alone (D), HAIC + TKIs (E) and HAIC + TKIs + ICIs (F) in PSM cohort. CI, confidence interval; HR. hazard ratio; PSM, propensity score matching.

**Additional file 5: Figure S3.** Forest plot of factors associated with OS (A) and PFS (B) in PSM cohort treated with HAIC 1d versus HAIC 2d. PSM, propensity score matching.

## Data Availability

The datasets used and/or analyzed during the current study are available from the corresponding author on reasonable request.

## Abbreviations

AFP: Alpha-fetoprotein
ALB: Albumin
ALBI: Albumin-bilirubin
ALT: Alanine aminotransferase
AST: Aspartate aminotransferase
BCLC: Barcelona Clinic Liver Cancer
CR: Complete response
DCR: Disease control rate
HbsAg: Hepatitis B surface antigen
HVTT: Hepatic vein tumor thrombus
ICIs: Immune checkpoint inhibitors
mRECIST: Modified Response Evaluation Criteria in Solid Tumors
NA: Not assessable
ORR: Objective response rate
PD: Progressive disease
PR: Partial response
PSM: Propensity score matching
PVTT: Portal vein invasion
RECIST: Response Criteria in Solid Tumors
SD: Stable disease
TBIL: Total bilirubin
TKIs: Tyrosine kinase inhibitors
