# Supplementary material for: One day versus two days of hepatic arterial infusion with oxaliplatin and fluorouracil for patients with unresectable hepatocellular carcinoma
Source: BMC Med. 2022 Oct 31;20:415. doi: 10.1186/s12916-022-02608-6 (PMC9620590; doi:10.1186/s12916-022-02608-6)
Supplement: Supplementary file 1 — Additional file 1. Methods. The details of ELISA. [file 12916_2022_2608_MOESM1_ESM.docx]

**Additional Methods**

**ELISA**

Peripheral blood samples were collected before the treatment. Five milliliters of whole blood were collected from each patient. Blood samples were then stood at room temperature for approximately 30 minutes until clotted. The clotted samples were centrifuged for 10 minutes at 1000 g. Approximately 500 µl of serum was collected and frozen at -80 °C. The Human TYMS ELISA Kit (Catalog No. EH15429) was purchased from FineTest. Before quantitative detection, the peripheral serum was placed at room temperature for 2 hours and centrifuged for 20 minutes at approximately 1000×g. After washing the plate 2 times, 100 µl of standards was added, and the sample was properly diluted into the test sample wells. Then, the plate was sealed with a cover and incubated at 37 °C for 90 minutes. The cover was removed, the plate content was discarded, and the plate was washed 2 times with wash buffer. Then, 100 µl of biotin-labeled antibody working solution was added to the above wells, and the plate was covered and incubated at 37 °C for 60 minutes. The cover was removed, the plate was washed 3 times with wash buffer, and the wash buffer was left in the wells for 2 minutes each time. Then, 100 µl of SABC working solution was added to each well, and the plate was covered and incubated at 37 °C for 30 minutes. The cover was removed, the plate was washed 5 times with wash buffer, and the wash buffer was left in the wells for 2 minutes each time. Then, 90 µl of TMB substrate was added to each well, and the plate was covered and incubated at 37 °C in the dark for no more than 30 minutes. After adding 50 µl of stop solution to each well, we immediately read the O.D. absorbance at 450 nm in a microplate reader.
