# Supplementary material for: One day versus two days of hepatic arterial infusion with oxaliplatin and fluorouracil for patients with unresectable hepatocellular carcinoma
Source: BMC Med. 2022 Oct 31;20:415. doi: 10.1186/s12916-022-02608-6 (PMC9620590; doi:10.1186/s12916-022-02608-6)
Supplement: Supplementary file 2 — Additional file 2: Table S1. Treatment administration. Table S2. Univariate and multivariate analysis of overall survival and progression-free survival. Table S3. Univariate and multivariate analysis of overall survival and progression-free survival in the PSM cohort. Table S4. Intrahepatic tumor response. Table S5. Baseline characteristics and tumor response within patients performing ELISA detection. [file 12916_2022_2608_MOESM2_ESM.docx]

Table S1. Treatment administration

|  | HAIC 2d (n=120) | HAIC 1d (n=248) | *p* |
| --- | --- | --- | --- |
| HAIC treatment |  |  |  |
| Mean (SD) | 3.45 (1.75) | 3.38 (1.77) | 0.73 |
| Median (IQR) | 3 (2-4) | 4 (2-4) | 0.81 |
| Subsequent treatment |  |  |  |
| Resection | 16 | 32 | 0.91 |
| Ablation | 6 | 19 | 0.34 |
| TACE | 15 | 33 | 0.83 |
| Radiotherapy | 5 | 11 | 0.26 |
| Sorafenib | 13 | 29 | 0.81 |
| Lenvatinib | 3 | 12 | 0.43 |
| PD-1 antibody | 1 | 8 | 0.30 |

Patients in the treatment groups receive subsequent PD-1 antibody such as nivolumab, pembrolizumab, toripalimab, sintilimab.

Table S2. Univariate and multivariate analysis of overall survival and progression-free survival

|  | Overall survival | | | Progression-free survival | | | |
| --- | --- | --- | --- | --- | --- | --- | --- |
|  | Univariate analysis | Multivariate analysis | | Univariate analysis | Multivariate analysis | | |
|  | *p*1 | HR (95%CI) | *p*2 | *p*1 | HR (95%CI) | *p*2 | |
| Group (HAIC 1d vs HAIC 2d) | 0.46 | - | - | 0.91 |  |  | |
| Age (>50 vs ≤50) | 0.008 | 0.86 (0.67-1.1) | 0.24 | 0.014 | 0.88 (0.70-1.1) | 0.30 | |
| Sex (Male vs Female) | 0.062 |  |  | 0.21 |  |  |  |
| HbsAg (Positive vs Negative) | 0.068 |  |  | 0.55 |  |  |  |
| Tumor size (>10cm vs ≤10cm) | 0.003 | 1.3 (0.99-1.63) | 0.062 | 0.07 |  |  |  |
| Tumor number (>3 vs ≤3) | <0.001 | 1.5 (1.1-1.9) | 0.005 | <0.001 | 1.4 (1.1-1.8) | 0.008 |  |
| PVTT （Vp3-4 vs Vp1-2&No） | <0.001 | 1.6 (1.3-2.0) | <0.001 | <0.001 | 1.3 (1.0-1.6) | 0.052 |  |
| HVTT (presence vs absence) | <0.001 | 1.2 (0.91-1.6) | 0.18 | 0.013 | 1.1 (0.86-1.5) | 0.38 |  |
| Metastasis (presence vs absence) | <0.001 | 1.9 (1.5-2.5) | <0.001 | <0.001 | 1.9 (1.4-2.4) | <0.001 |  |
| AFP (>400 vs ≤400) | <0.001 | 1.4 (1.1-1.7) | 0.016 | 0.037 | 1.2 (0.93-1.5) | 0.18 |  |
| PIVKA-II (>8286 vs ≤8286) | 0.045 | 1.3 (0.91-1.5) | 0.24 | 0.33 |  |  |  |
| CA199 (>30.9 vs ≤30.9) | 0.084 |  |  | 0.53 |  |  |  |
| ALBI (Grade 2 vs Grade 1) | <0.001 | 1.5 (1.2-1.9) | <0.001 | 0.008 | 1.2 (0.97-1.5) | 0.084 |  |

Abbreviations: ALBI, albumin-bilirubin

P1 value was calculated with two-sided log-rank test. Any factors that were statistically significant at P<0.05 in the univariate analysis were candidates for entry into a multivariable Cox analysis.

P2 value was calculated by multivariable Cox proportional-hazards analysis.

Table S3. Univariate and multivariate analysis of overall survival and progression-free survival in the PSM cohort

|  | Overall survival | | | Progression-free survival | | | |
| --- | --- | --- | --- | --- | --- | --- | --- |
|  | Univariate analysis | Multivariate analysis | | Univariate analysis | Multivariate analysis | | |
|  | *p*1 | HR (95%CI) | *p*2 | *p*1 | HR (95%CI) | *p*2 | |
| Group (HAIC 1d vs HAIC 2d) | 0.35 | - | - | 0.87 |  |  | |
| Age (>50 vs ≤50) | 0.11 |  |  | 0.026 | 0.84 (0.62-1.1) | 0.24 | |
| Sex (Male vs Female) | 0.047 | 1.7 (0.95-2.9) | 0.076 | 0.19 |  |  | |
| HbsAg (Positive vs Negative) | 0.024 | 2 (1.2-3.5) | 0.013 | 0.85 |  |  | |
| Tumor size (>10cm vs ≤10cm) | 0.02 | 1.2 (0.90-1.7) | 0.19 | 0.19 |  |  | |
| Tumor number (>3 vs ≤3) | <0.001 | 1.5 (1.0-2.0) | 0.035 | <0.001 | 1.6 (1.1-2.2) | 0.008 | |
| PVTT （Vp3-4 vs Vp1-2&No） | <0.001 | 1.3 (0.98-1.8) | 0.068 | 0.016 | 1.4 (1.0-1.9) | 0.037 | |
| HVTT (presence vs absence) | <0.001 | 1.5 (1.0-2.2) | 0.041 | 0.003 | 1.6 (1.1-2.3) | 0.01 | |
| Metastasis (presence vs absence) | <0.001 | 1.9 (1.4-2.7) | <0.001 | <0.001 | 1.8 (1.3-2.5) | <0.001 | |
| AFP (>400 vs ≤400) | 0.008 | 1.5 (1.1-2.1) | 0.01 | 0.055 |  |  | |
| PIVKA-II (>8286 vs ≤8286) | 0.043 | 1.4 (0.7-1.3) | 0.83 | 0.41 |  |  |  |
| CA199 (>30.9 vs ≤30.9) | 0.15 |  |  | 0.57 |  |  |  |
| ALBI (Grade 2 vs Grade 1) | <0.001 | 1.4 (1.1-2.0) | 0.023 | 0.097 |  |  | |

Abbreviations: ALBI, albumin-bilirubin; PSM, propensity score matching

P1 value was calculated with two-sided log-rank test. Any factors that were statistically significant at P<0.05 in the univariate analysis were candidates for entry into a multivariable Cox analysis.

P2 value was calculated by multivariable Cox proportional-hazards analysis.

Table S4. Intrahepatic tumor response

|  | RECIST 1.1 | |  |  | mRECIST | |  |
| --- | --- | --- | --- | --- | --- | --- | --- |
|  | HAIC 2d | HAIC 1d | *p*^a^ |  | HAIC 2d | HAIC 1d | *p*^a^ |
| CR | 0 | 0 |  |  | 10 (8.3%) | 15 (6.0%) | 0.41 |
| PR | 55 (45.8%) | 103 (41.5%) | 0.44 |  | 62 (51.7%) | 118 (47.6%) | 0.46 |
| SD | 37 (30.8%) | 84 (33.9%) | 0.56 |  | 23 (19.2%) | 59 (23.8%) | 0.32 |
| PD | 18 (15%) | 39 (15.7%) | 0.86 |  | 15 (12.5%) | 34 (13.7%) | 0.75 |
| NA | 10 (8.3%) | 22 (8.9%) | 0.86 |  | 10 (8.3%) | 22 (8.9%) | 0.86 |
| ORR | 55 (45.8%) | 103 (41.5%) | 0.44 |  | 72 (60.0%) | 133 (53.6%) | 0.25 |
| DCR | 92 (76.7%) | 187 (75.4%) | 0.79 |  | 95 (79.2%) | 192 (77.4%) | 0.71 |
|  | RECIST 1.1 | |  |  | mRECIST | |  |
| PSM cohort | HAIC 2d | HAIC 1d | *p*^a^ |  | HAIC 2d | HAIC 1d | *p*^a^ |
| CR | 0 | 0 |  |  | 10 (8.8%) | 5 (4.4%) | 0.18 |
| PR | 49 (43.4%) | 46 (40.7%) | 0.69 |  | 56 (49.6%) | 59 (52.2%) | 0.69 |
| SD | 37 (32.7%) | 41 (36.3%) | 0.58 |  | 30 (26.5%) | 24 (21.2%) | 0.35 |
| PD | 17 (15%) | 14 (12.4%) | 0.56 |  | 15 (13.3%) | 13 (11.5%) | 0.69 |
| NA | 10 (8.8%) | 12 (10.6%) | 0.65 |  | 10 (8.8%) | 12 (10.6%) | 0.65 |
| ORR | 49 (43.4%) | 46 (40.7%) | 0.69 |  | 66 (58.4%) | 64(56.6%) | 0.79 |
| DCR | 86 (76.1%) | 87 (77%) | 0.88 |  | 96 (85%) | 88 (77.9%) | 0.17 |

Abbreviations: CR, complete response; DCR, disease control rate; mRECIST, modified Response Evaluation Criteria in Solid Tumors; NA, not assessable; ORR, objective response rate; PD, progressive disease; PR, partial response; RECIST, Response Criteria in Solid Tumors; SD, stable disease.

^a^Statistical significance was assessed with the Chi-square test.

Table S5. Baseline characteristics and tumor response within patients performing ELISA detection

|  | HAIC 2d (n=88) | HAIC 1d (n=215） | *p* |
| --- | --- | --- | --- |
| Age, year, mean (SD) | 50 (11.1) | 50 (11.8) | 0.90 |
| ≤50 | 44 | 117 | 0.97 |
| ＞50 | 44 | 108 |  |
| Sex |  |  | 0.50 |
| male | 78 | 196 |  |
| female | 10 | 19 |  |
| ALB, median (IQR), g/dL | 40.8 (37.3-44.3) | 40.3 (37.8-43.5) | 0.55 |
| ALT, median (IQR), U/L | 43.9 (28.8-70.0) | 45.8 (31.9-69.2) | 0.41 |
| AST, median (IQR), U/L | 68.2 (48.1-111.4) | 60.3 (43.1-101.7) | 0.25 |
| TBIL, median (IQR), µmol/L | 16.3 (12.4-22.2) | 15.9 (12.1-21.8) | 0.84 |
| PT, median (IQR), s | 12.6 (11.6-13.6) | 12.4 (11.7-13.0) | 0.25 |
| Tumor size, cm, median (IQR) | 9.9 (7.5-12.8) | 10.2 (7.8-13.1) | 0.55 |
| ≤10 | 45 | 104 | 0.66 |
| ＞10 | 43 | 111 |  |
| Tumor number |  |  | 0.57 |
| ≤3 | 35 | 78 |  |
| ＞3 | 53 | 137 |  |
| PVTT (Japan) |  |  | 0.85 |
| Vp1-2&No | 53 | 132 |  |
| Vp3-4 | 35 | 83 |  |
| DVTT |  |  | 0.88 |
| No | 74 | 176 |  |
| Hepatic vein | 9 | 24 |  |
| Inferior vena cava | 5 | 15 |  |
| Extrahepatic metastasis |  |  | 0.32 |
| No | 68 | 160 |  |
| Organ only | 7 | 20 |  |
| Lymph node only | 12 | 23 |  |
| Both | 1 | 12 |  |
| AFP, ng/ml |  |  |  |
| ≤400 | 33 | 89 | 0.53 |
| ＞400 | 55 | 126 |  |
| BCLC stage  A or B  C  TYMS, median(IQR), ng/μl  High  low  CR&PR (RECIST 1.1)  CR&PR (mRECIST) | 31  57  3.6 (2.5-5.4)  38  50  40  46 | 74  141  4.1 (2.9-5.7)  113  102  87  91 | 0.89  0.16  0.14  0.51  0.15 |

Abbreviations: CR, complete response; mRECIST, modified Response Evaluation Criteria in Solid Tumors; PR, partial response; RECIST, Response Criteria in Solid Tumors.
