# Supplementary figures and images for: One day versus two days of hepatic arterial infusion with oxaliplatin and fluorouracil for patients with unresectable hepatocellular carcinoma
Source: BMC Med. 2022 Oct 31;20:415. doi: 10.1186/s12916-022-02608-6 (PMC9620590; doi:10.1186/s12916-022-02608-6)

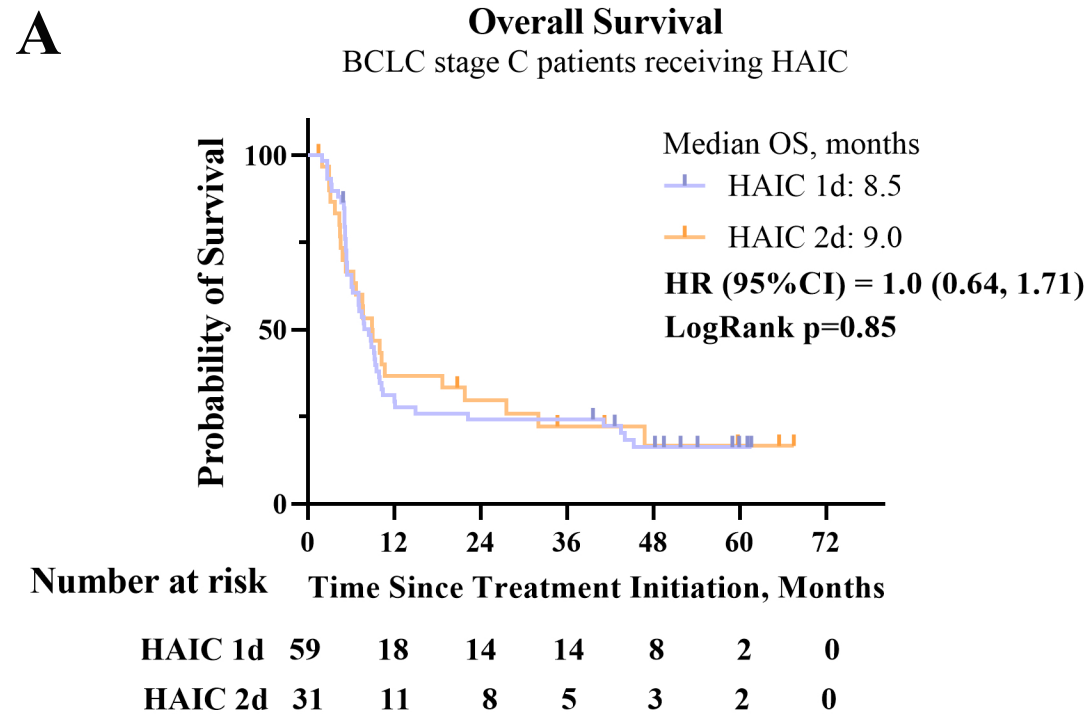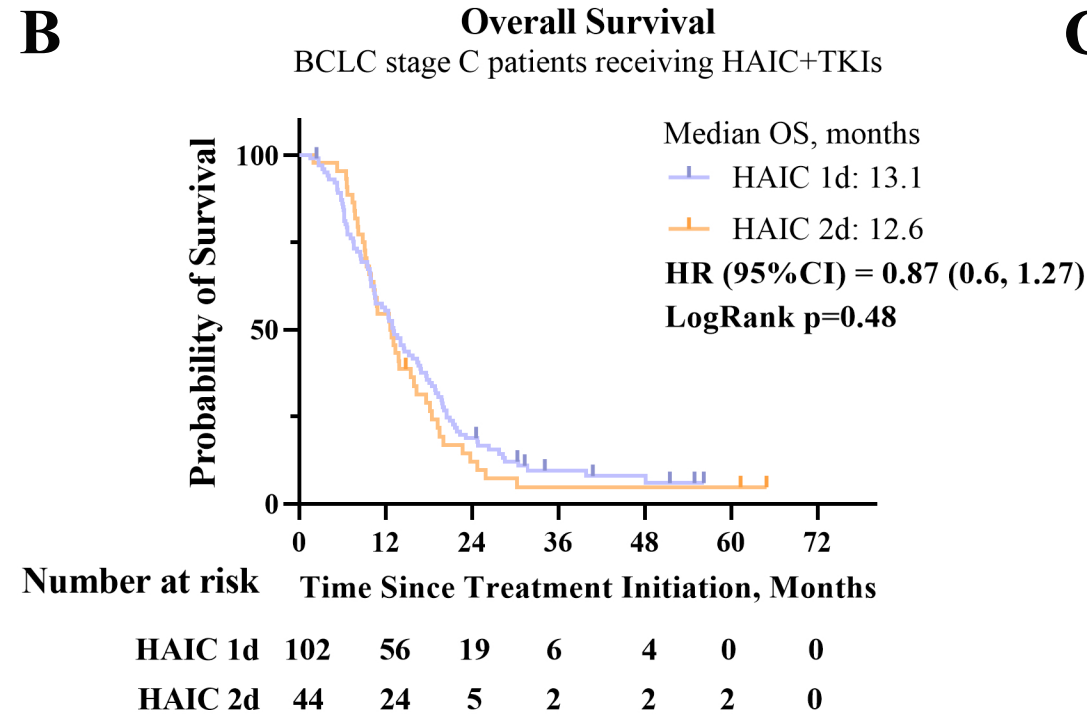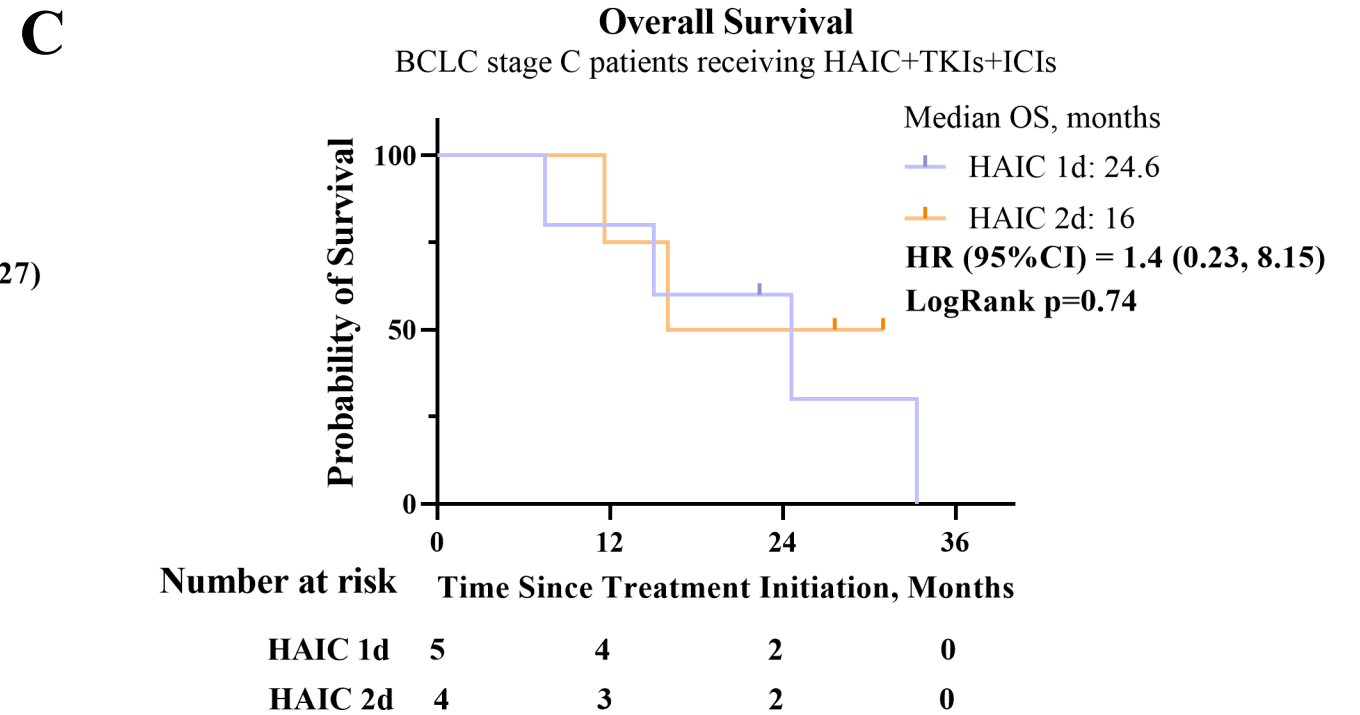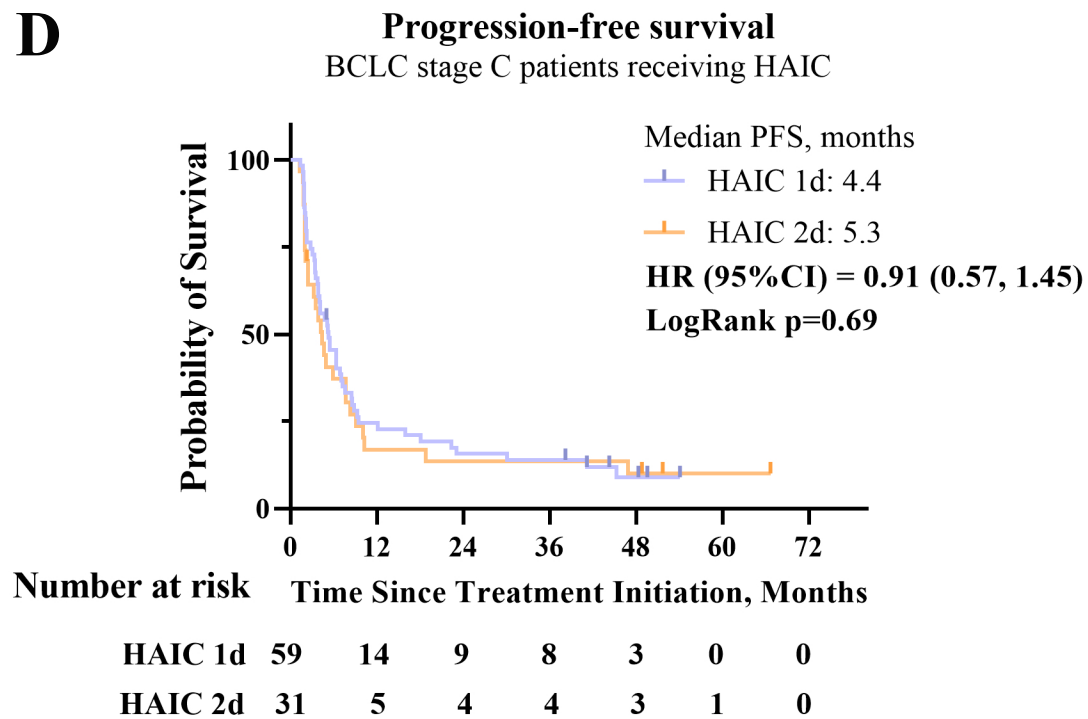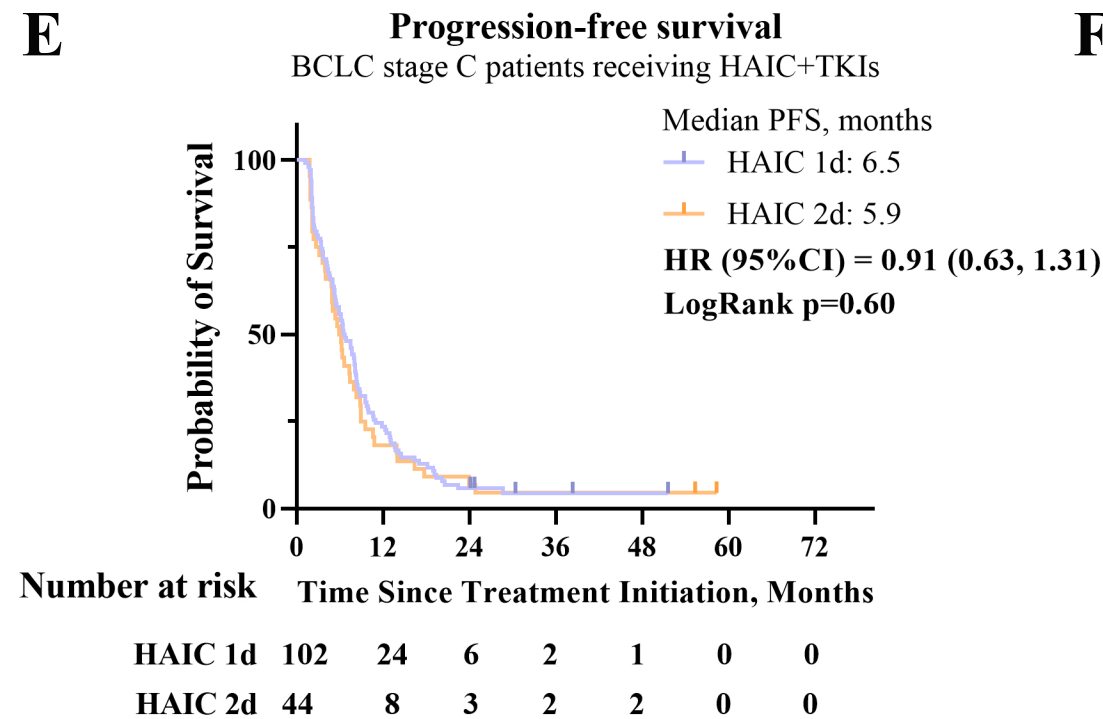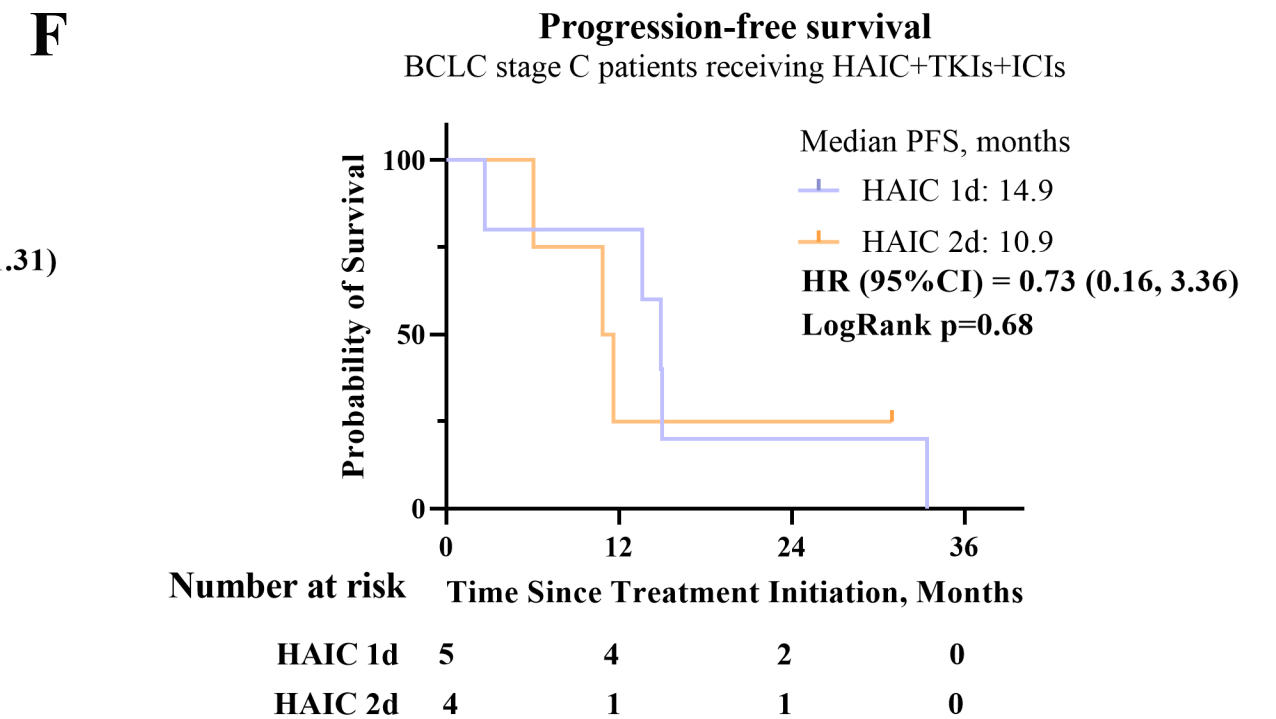

Supplement: Supplementary file 3 — Additional file 3: Figure S1. Kaplan-Meier curves of overall survival in BCLC stage C patients receiving HAIC alone (A), HAIC + TKIs (B) and HAIC + TKIs + ICIs (C). Kaplan-Meier curves of progression-free survival in BCLC stage C patients receiving HAIC alone (D), HAIC + TKIs (E) and HAIC + TKIs + ICIs (F). CI, confidence interval; HR. hazard ratio. [file 12916_2022_2608_MOESM3_ESM.pdf]

A

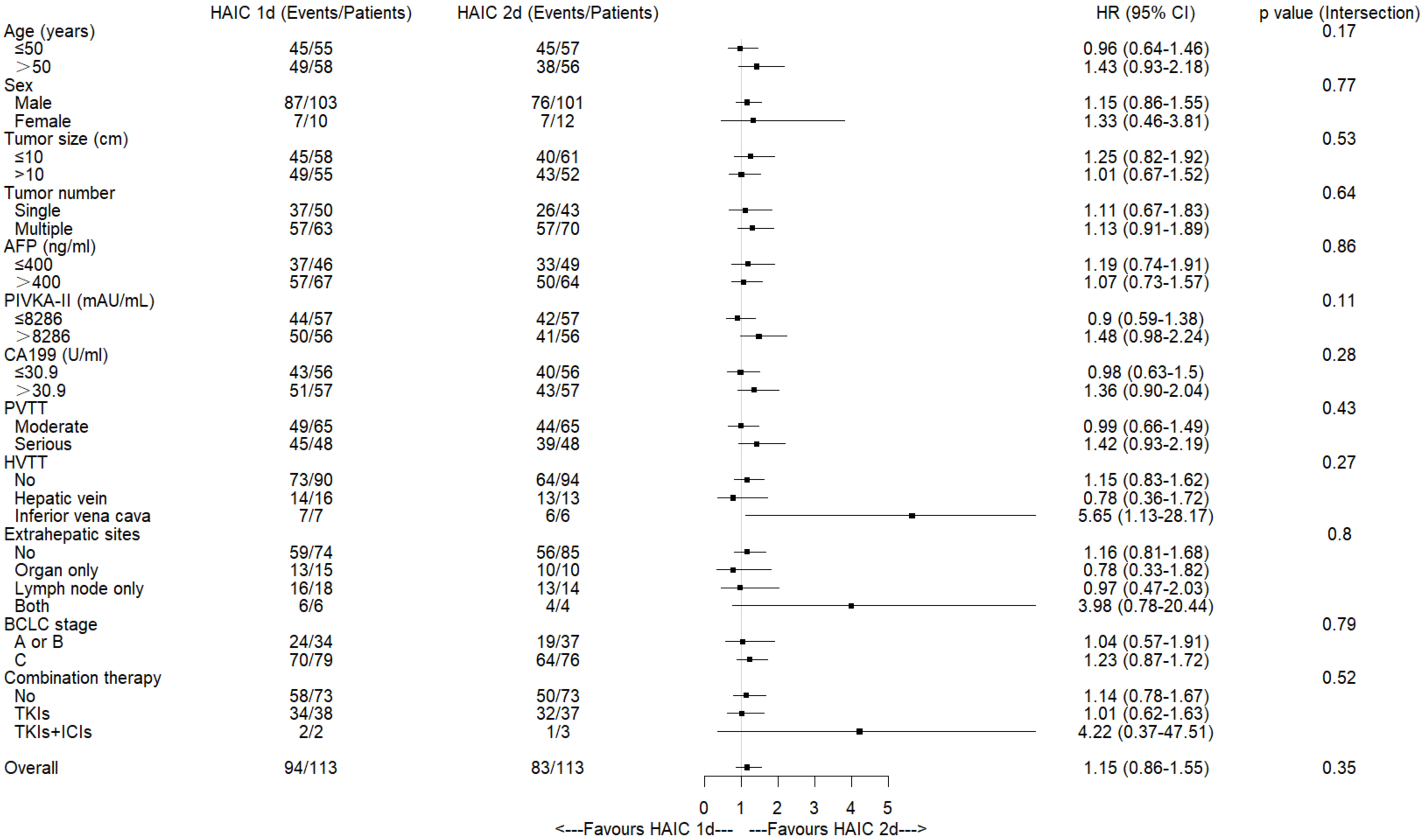

B

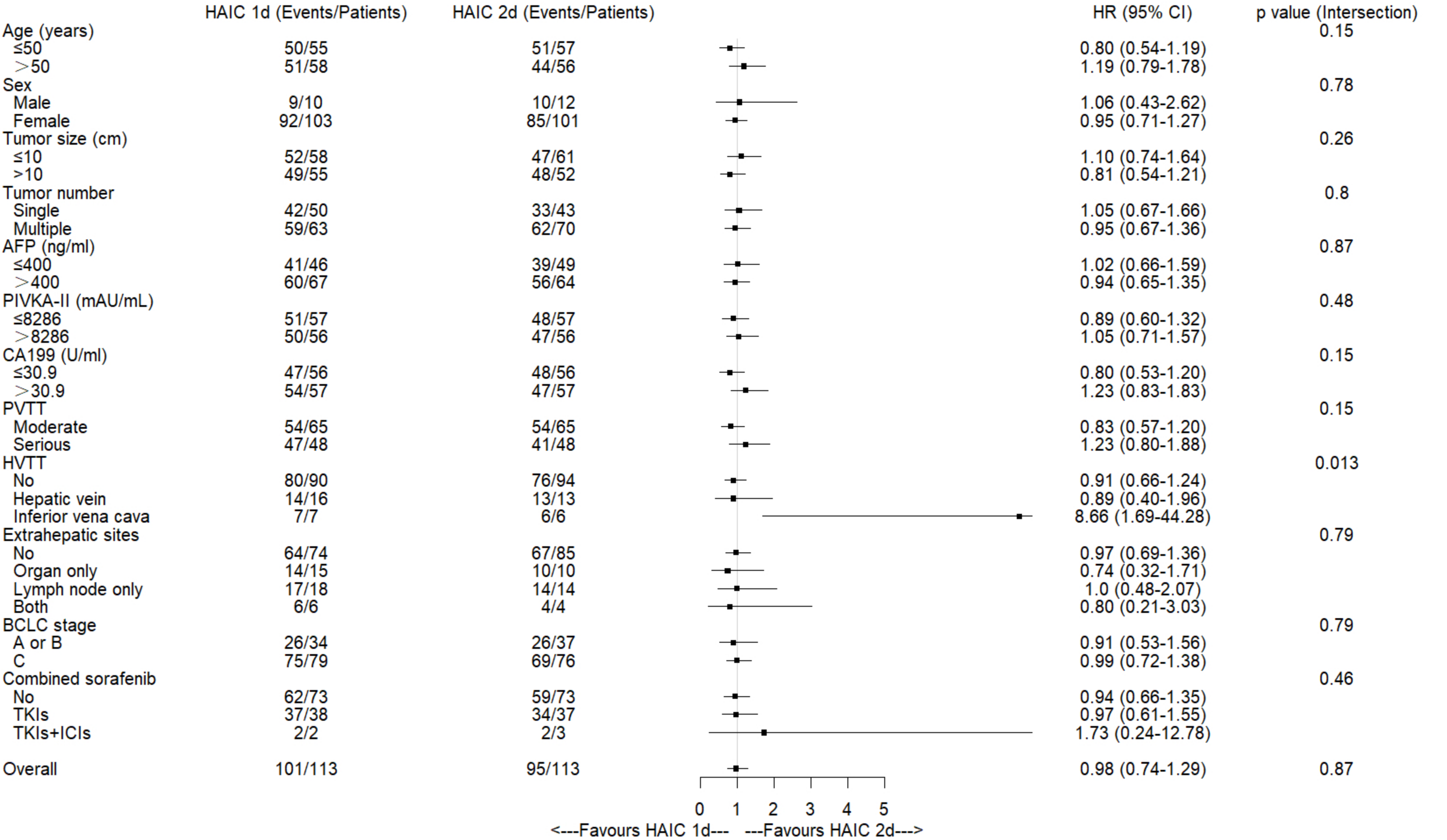

Supplement: Supplementary file 5 — Additional file 5: Figure S3. Forest plot of factors associated with OS (A) and PFS (B) in PSM cohort treated with HAIC 1d versus HAIC 2d. PSM, propensity score matching. [file 12916_2022_2608_MOESM5_ESM.pdf]
