# Supplementary material for: One day versus two days of hepatic arterial infusion with oxaliplatin and fluorouracil for patients with unresectable hepatocellular carcinoma
Source: BMC Med. 2022 Oct 31;20:415. doi: 10.1186/s12916-022-02608-6 (PMC9620590; doi:10.1186/s12916-022-02608-6)
Supplement: Supplementary file 4 — Additional file 4: Figure S2. Kaplan-Meier curves of overall survival in BCLC stage C patients receiving HAIC alone (A), HAIC + TKIs (B) and HAIC + TKIs + ICIs (C) in PSM cohort. Kaplan-Meier curves of progression-free survival in BCLC stage C patients receiving HAIC alone (D), HAIC + TKIs (E) and HAIC + TKIs + ICIs (F) in PSM cohort. CI, confidence interval; HR. hazard ratio; PSM, propensity score matching. [file 12916_2022_2608_MOESM4_ESM.pdf]

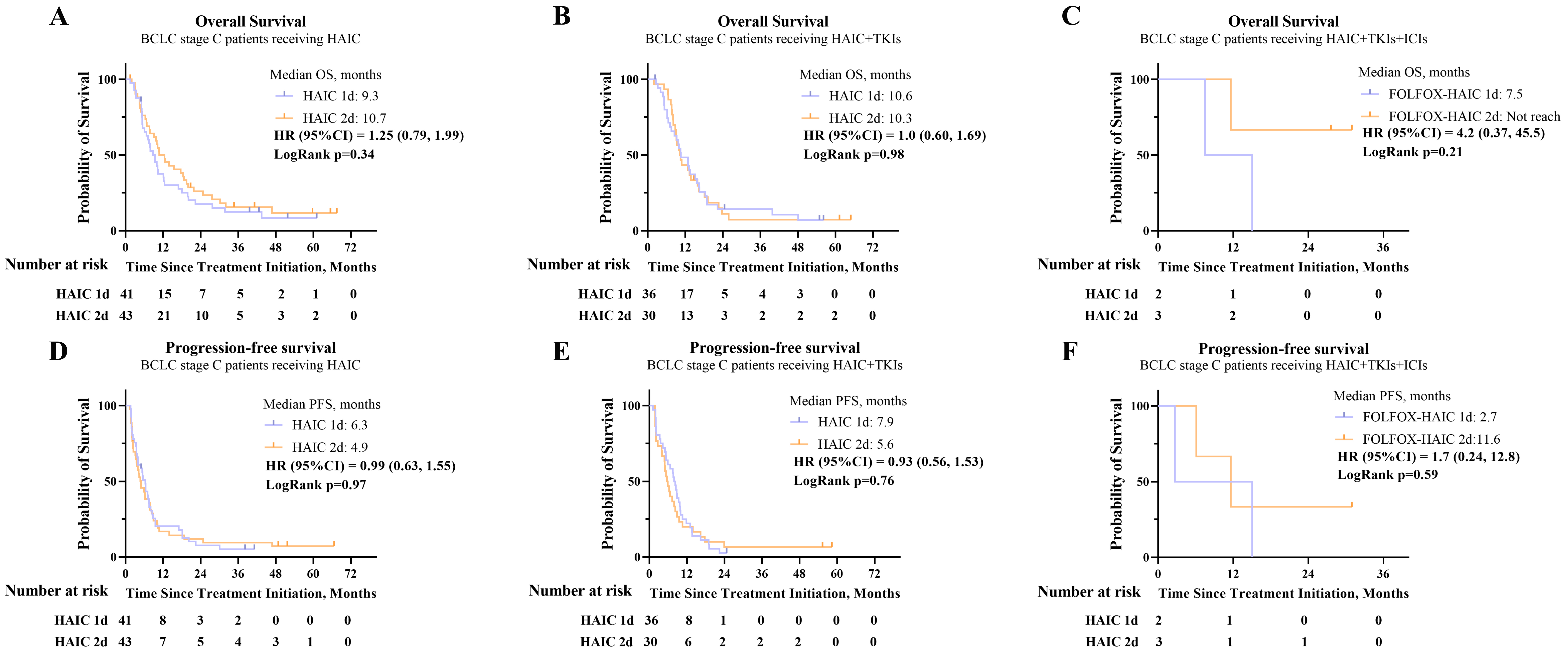
A
Overall Survival
BCLC stage C patients receiving HAIC
Median OS, months
HAIC 1d: 9.3
HAIC 2d: 10.7
HR (95%CI) = 1.25 (0.79, 1.99)
LogRank p=0.34
Probability of Survival
Time Since Treatment Initiation, Months
Number at risk
HAIC 1d 41 15 7 5 2 1 0
HAIC 2d 43 21 10 5 3 2 0
B
Overall Survival
BCLC stage C patients receiving HAIC+TKIs
Median OS, months
HAIC 1d: 10.6
HAIC 2d: 10.3
HR (95%CI) = 1.0 (0.60, 1.69)
LogRank p=0.98
Probability of Survival
Time Since Treatment Initiation, Months
Number at risk
HAIC 1d 36 17 5 4 3 0 0
HAIC 2d 30 13 3 2 2 2 0
C
Overall Survival
BCLC stage C patients receiving HAIC+TKIs+ICIs
Median OS, months
FOLFOX-HAIC 1d: 7.5
FOLFOX-HAIC 2d: Not reach
HR (95%CI) = 4.2 (0.37, 45.5)
LogRank p=0.21
Probability of Survival
Time Since Treatment Initiation, Months
Number at risk
HAIC 1d 2 1 0 0
HAIC 2d 3 2 0 0
D
Progression-free survival
BCLC stage C patients receiving HAIC
Median PFS, months
HAIC 1d: 6.3
HAIC 2d: 4.9
HR (95%CI) = 0.99 (0.63, 1.55)
LogRank p=0.97
Probability of Survival
Time Since Treatment Initiation, Months
Number at risk
HAIC 1d 41 8 3 2 0 0 0
HAIC 2d 43 7 5 4 3 1 0
E
Progression-free survival
BCLC stage C patients receiving HAIC+TKIs
Median PFS, months
HAIC 1d: 7.9
HAIC 2d: 5.6
HR (95%CI) = 0.93 (0.56, 1.53)
LogRank p=0.76
Probability of Survival
Time Since Treatment Initiation, Months
Number at risk
HAIC 1d 36 8 1 0 0 0 0
HAIC 2d 30 6 2 2 2 0 0
F
Progression-free survival
BCLC stage C patients receiving HAIC+TKIs+ICIs
Median PFS, months
FOLFOX-HAIC 1d: 2.7
FOLFOX-HAIC 2d:11.6
HR (95%CI) = 1.7 (0.24, 12.8)
LogRank p=0.59
Probability of Survival
Time Since Treatment Initiation, Months
Number at risk
HAIC 1d 2 1 0 0
HAIC 2d 3 1 1 0
